# Hemoglobin Regeneration Efficiency and Relative Iron Bioavailability of Four Elemental Iron Powders in Rats

**DOI:** 10.3390/nu16142258

**Published:** 2024-07-13

**Authors:** James H. Swain, Ryan C. Nemeth, Anshul R. Bethi, Caroline J. Jang, Eva L. Zheng

**Affiliations:** 1 Department of Nutrition, School of Medicine, Case Western Reserve University, 10900 Euclid Avenue, Cleveland, OH 44106, USA; 2 Scientific Enrichment Opportunity Program, School of Medicine, Case Western Reserve University, 10900 Euclid Avenue, Cleveland, OH 44106, USA

**Keywords:** elemental iron powder, fortification, absorption, relative bioavailability, hemoglobin regeneration efficiency

## Abstract

Effective food fortification strategies using elemental iron powders (EIPs) are needed to combat iron deficiency anemia. The purpose of this study was to determine hemoglobin regeneration efficiency (HRE) and relative iron bioavailability (RBV) of four food-grade EIPs (El-Lyte (EL), Hi-Sol (HS), H-325 (H3), and A-131 (A1)) by treating anemic rats with 14 d iron repletion diets (uncooked and cooked), fortified with a 12, 24, or 36 mg iron/kg diet of the EIPs, ferrous sulfate monohydrate (FS, FeSO_4_•H_2_O), or no added iron (control), n = 9–12/group. The ability of EL and HS to maintain hemoglobin for 6 weeks on the 6 mg iron/kg diet was also studied. The dissolution rate of iron from the EIPs was measured in hydrochloric acid at pH 1.0. Compared to FS, the EL, HS, and A1 EIPs had >50% overall RBV, with the following order: HS > A1 > EL > H3 (*p* ≤ 0.05); the effect of cooking was not significant (*p* > 0.05). Dissolution testing revealed that the mean RBV of the EIPs was positively associated with the percentage of iron solubility. In the 6-week maintenance study, EL and HS maintained hemoglobin as well as FS. Overall, the findings show that at the concentrations of iron tested, these EIPs are effective fortification agents to replenish hemoglobin and correct iron deficiency anemia.

## 1. Introduction

Elemental iron powders (EIPs) are often used to fortify staple foods because they are a cost-effective approach to reducing iron deficiency anemia, the most prevalent micronutrient deficiency worldwide [1]. EIPs are often preferred as fortification agents because they do not promote unfavorable sensory or organoleptic changes in food during storage. However, the ability of many EIPs that are commonly used to fortify foods to improve iron status has not been thoroughly studied [2]. Furthermore, methods used to produce EIPs vary among manufacturers, resulting in physicochemical differences [3], and evidence suggests that such variation may influence iron bioavailability [4,5]. Additional information regarding the comparative nutritional usefulness of EIPs would enhance ongoing food fortification programs worldwide [6].

An expert panel reviewing animal [4,5,6,7,8,9,10,11,12] and human studies [6,13,14,15,16,17,18,19,20,21] concluded that there is inadequate information regarding the types of EIPs currently being used to fortify staple foods [22]. Historically, EIPs have comprised the majority of iron used to fortify foods since the late 1980s [23,24]. Prior studies of nutrient efficacy have included the assessment of iron absorption using rats as an in vivo model to determine hemoglobin regeneration efficiency (HRE) and the relative bioavailability (RBV, compared to ferrous sulfate, a highly bioavailable form of iron) of a micronutrient [11], and a previous review study found excellent agreement between human tracer studies and rat hemoglobin repletion [12]. As new EIPs enter the market for use as food fortificants, thorough testing of the ability of these new agents and the impact they may have on improving iron status globally is needed.

The purpose of this study was to determine the HRE and RBV of four food-grade elemental iron powders, the effect of uncooked vs. cooked diets on RBV, and the association between these elemental iron powders’ solubility and their RBVs.

## 2. Materials and Methods

### 2.1. Elemental Iron Powders

In this study, four food-grade elemental iron powders (El-Lyte (EL, an electrolytic iron powder), Hi-Sol (HS, a novel iron powder), H-325 (H3, a hydrogen-reduced iron powder), and A-131 (A1, a well-known standard food-grade electrolytic iron powder) (North American Höganas, Inc., Somerset County, PA, USA) were tested. All of these elemental iron powders (EIPs) meet Food Chemical Codex (FCC) specifications [25]. The EIPs were incorporated into diets that were uncooked or cooked. Bakery-grade ferrous sulfate monohydrate (FS, FeSO_4_•H_2_O, Crown Technologies, Inc, Indianapolis, IN, USA), an iron salt known for its highly absorbable non-heme iron, also in uncooked and cooked diets, was used as a positive control and for comparisons to determine relative iron bioavailability (RBV). A “no added iron” diet served as the standard control. EIPs and FS were stored in a desiccator under vacuum at room temperature (72 °F) until use. All four EIPs (EL, HS, H3, and A1) are considered “elemental iron powders”, referring to the final chemical form or compound, which is relatively pure elemental iron (>98% iron *w*/*w*, zero oxidation state, with typically >95% of the particle sizes <45 µm diameter). The apparent densities (g/cm^3^) of the EIPs are as follows: EL = 1.96, HS = 1.77, H3 = 1.97, and A1 = 2.03. Although structurally different (i.e., flake-like vs. granular for electrolytic vs. H-reduced, respectively), >98% of all four EIPs’ particles pass through a +325 mesh sieve.

### 2.2. Study Design and Dietary Treatments

An initial 432 weanling male Sprague-Dawley rats (Charles River/SASCO, Wilmington, MA, USA) were used. Rats were housed individually in wire-bottom stainless steel mesh cages in a room controlled for temperature (21 ± 1 °C), humidity, and given a 12 h light:dark cycle. After 24 d of depletion with an iron-deficient diet (1.6 mg iron/kg AIN-93G diet; approximately 1.4 mg iron/kg diet based on analyzed iron content), iron-deficient rats with hemoglobin values between 3 and 6 g/dL (mean ± SEM of 4.1 ± 0.4 g/dL; range 3.1–5.9 g/dL) were then randomly assigned by blocking on hemoglobin to one of 36 different repletion-period diet groups. Rats consumed the repletion diets, uncooked or cooked, for 14 d, fortified with EL, HS, H3, or A1 (at 12, 24, or 36 mg iron/kg diet), FS (at 6, 12, 18, 24, or 36 mg iron/kg diet), or no added iron, n = 9–12/group. During the repletion period, daily and total food consumption was measured, including adjustments for spilled food. All diets and deionized, distilled water were fed ad libitum. The HRE ratio and RBV calculations account for iron intake and body weight. All animal procedures followed the Institutional Animal Care and Use Committee procedures at Case Western Reserve University (CWRU), in accordance with the NIH guidelines.

The four EIPs or FS were incorporated into a phytate-free diet modified to have a very low base iron content, using vitamin-free casein (Harlan Teklad, Madison, WI, USA) and a high-purity cellulose fiber source (Alphacel™; ICN Biomedicals, Irvine, CA, USA). The diet pH was neutral (7.0). The base modified diet [26] (AIN-93G*[M]) composition, from which treatment (repletion) diets were prepared, is shown in Table 1. Without added iron, the diet contained approximately 1.5 mg iron/kg diet according to the analysis. EIPs and FS were added into diets taking into account the baseline amount (analyzed) already present in the control (no added iron) group, uncooked and cooked. The modified mineral mix omitted ferric citrate, and the vitamin mix used considered calcium and ascorbic acid content (AIN-93-VX; Harlan Teklad, Madison, WI, USA).

Mixing of each EIP and FS into repletion-period diets and analysis of each diet’s iron content were performed in a similar manner as previously described [27], with modifications. Briefly, before being added to the rest of the diet, each EIP or FS was combined with a 500 g portion of corn starch. After thoroughly mixing as previously described [27], the corn starch/iron powder mixture was then added to the other dry diet ingredients and mixed (D330 mixer; Hobart, Troy, OH, USA) for 30 min. Oil was then added, and the diet was mixed for an additional 30 min, followed by the addition of 10% v/w distilled water; this percentage of added water represents a typical flour–water mixture often used in flour-based prepared foods. A concurrent (matching) diet was prepared in this manner and then cooked in a convection oven (model VSOF7301; Viking Inc., Greenwood, MS, USA) at 375 °F (190 °C) for 25 min. Diets were then vacuum-packed (Pro-2100 Vacuum Sealer; Westin, Inc., Southern Pines, NC, USA) and stored in the dark at 39 °F until use. A small portion of each uncooked and cooked diet was taken for analysis to confirm the iron content, as previously described [27].

### 2.3. Iron Powder Dissolution (Solubility) Testing

The dissolution (solubility) of iron from the elemental iron powder particles was measured using a method similar to that previously described [6] using analytical-grade 18.0 ΩM-cm water (Nanopure, Model D11900 series; ThermoFisher Scientific, Dubuque, IA, USA). After placing 20 mg of each elemental iron powder in 250 mL of dilute 0.1 mol/L hydrochloric acid at pH 1.0, the solution was immediately subjected to constant orbital stirring (Model ORS 200; Boekel Scientific, Feasterville, PA, USA) at 150 rpm and 37 °C. No magnetic stirring devices or glass beads were used. Iron in the solution was then assayed at 30 min; these conditions (pH of 1.0 and sampling at 30 min) were previously found to be optimal [27]. At 30 min, stirring was stopped, and 1 mL of solution was quickly withdrawn and, within 30 sec, centrifuged (Bio-fugePico, 7500 series model; Heraeus Instruments, South Plainfield, NJ, USA) for 5 min at 11,600× *g*. Immediately after centrifugation, 500 µL of the supernatant was diluted to 5 mL with an additional 0.1 mol/L of hydrochloric acid. The concentration of iron in the solution was then analyzed through inductively coupled plasma–atomic emission spectroscopy (ICP-AES, Model ICPE-9800; Shimadzu Scientific Instruments, Kyoto, Japan). Solubility values are expressed as the mean ± SEM of triplicate samples determined via separate tests.

### 2.4. Hemoglobin and Hemoglobin Iron Determinations

Hemoglobin and hemoglobin iron determinations were performed similarly as previously described [27,28] but using tail vein blood collection. The following calculation was used to determine hemoglobin (Hb) iron:

Hb Fe (mg) = BW (kg) × 0.067 × Grams Hb per mL × 3.35 mg Fe

Note: the calculation assumes that 6.7% of body weight (BW; kg) is blood and the iron content of hemoglobin is 3.35 mg/g [8,9]. Therefore, hemoglobin iron was determined on the basis of 3.35 mg iron/g hemoglobin and 0.075 L blood/kg body weight [29].

Following repletion, the rats were anesthetized and sacrificed as previously described [27].

### 2.5. Hemoglobin Regeneration Efficiency and Relative Iron Bioavailability

The hemoglobin regeneration efficiency (HRE) of the four EIPs (EL, HS, H3, and A1) and FS and the relative biological value (relative iron bioavailability, RBV) of the four EIPs were determined as described previously [30]. The RBV of the EIPs was calculated as a percentage of HRE relative to the HRE of FS; HRE and RBV determinations were made by matching cooked vs. cooked and uncooked vs. uncooked data for the same concentration of iron in the diet (diet-matched) and using the analyzed value of iron for each diet. For RBV determinations, bakery-grade ferrous sulfate monohydrate (FS) was selected as the reference standard (positive control) because of its known and higher bioavailability and the prevalence of data in the literature [5,6]. HRE and RBV were calculated based on the following formulas:

HRE ratio = [Final Hb Fe (mg) − Initial Hb Fe (mg)]/Fe intake (mg total consumed; analyzed diet value)

RBV = Percentage (%) HRE relative to ferrous sulfate monohydrate (FS): HRE ratio of elemental iron powder/HRE ratio of the FS group × 100 (diet-matched)

### 2.6. Hemoglobin Maintenance Study Arm

In a separate arm of the study, EL and HS were added to uncooked and cooked diets to provide 6 mg Fe/kg diet to determine how well these two EIPs maintained iron status (hemoglobin) during a 6-week period. Hemoglobin measures were taken at baseline, mid-treatment (week 3), and at the end of the treatment period (week 6). Ninety-six rats (with normal hemoglobin, not anemic) were randomly assigned to one of eight treatment groups: control (no added iron), FS (positive control), EL, or HS, uncooked or cooked (n = 9–12/group).

### 2.7. Statistical Analyses

Power analyses were performed based on previous published data [27]. Data on repletion of hemoglobin concentration and change in total hemoglobin iron relative to dietary iron concentration (analyzed) from each iron source as well as absolute iron intake (mg/d) were analyzed as previously described [30]. FS served as the positive control for RBV determinations, and the “no added iron” group served as the overall control (blank), as previously described [31]. Dissolution data were analyzed using one-way analysis of variance (ANOVA), and differences among HRE and RBV means tested using Tukey’s multiple comparison and Duncan post hoc testing using the statistical package SAS (SAS Version 10.2, SAS Institute, Cary, NC, USA). Differences were considered significant if *p* ≤ 0.05. All values are expressed as mean ± SEM. Illustration of data and results was performed using GraphPad Prism (software version 10.2; GraphPad, Boston, MA, USA).

## 3. Results

### 3.1. Elemental Iron Powder Dissolution (Solubility)

The percentage of dissolution of iron from the four elemental iron powders is shown in Table 2. Dissolution (solubility) of iron from the EL, HS, H3, and A1 EIPs at 30 min in HCl solution (pH 1.0) at 37 °C was 58.7 ± 1.1, 75.5 ± 2.7, 43.7 ± 1.4, and 72.7 ± 3.0%, respectively. Figure S1 illustrates the differences in percentage of dissolution graphically.

### 3.2. Hemoglobin Change and Hemoglobin Regeneration Efficiency: No Added Iron and Ferrous Sulfate

**Figure 1:**
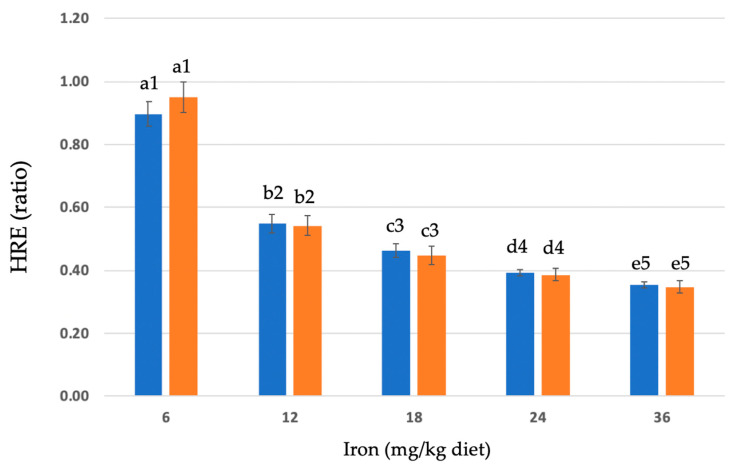
Hemoglobin (Hb) regeneration efficiency of ferrous sulfate monohydrate (FeSO_4_•H_2_O, FS) in uncooked and cooked diets (blue and orange bars, respectively). Values are mean ± SEM (n = 9–12/group). Different letters are used to denote significant differences (*p* ≤ 0.05) between different dietary iron (Fe) concentrations (mg Fe/kg diet) of the same treatment (uncooked and cooked). Different numbers are used to designate significant differences (*p* ≤ 0.05) between uncooked and cooked diets of the same Fe concentration. HRE ratio = [Final Hb Fe (mg) − Initial Hb Fe (mg)]/Fe intake (mg total consumed).

Hemoglobin change and hemoglobin regeneration efficiency (HRE) in anemic rats fed no added iron or bakery-grade ferrous sulfate monohydrate (FS) in uncooked or cooked diets are shown in Table 3. Food intake and, thus, iron intake and weight gain were positively associated with increasing dietary iron in all treatment groups (data in Table 3). The HRE ratio calculation accounts for iron intake. The HRE of FS in uncooked and cooked diets is shown in Figure 1. Figure S2 illustrates the hemoglobin change (g/dL) in the control and FS in uncooked and cooked diets, graphically.

### 3.3. Hemoglobin Regeneration Efficiency and Relative Iron Bioavailability of Elemental Iron Powders

**Figure 2:**
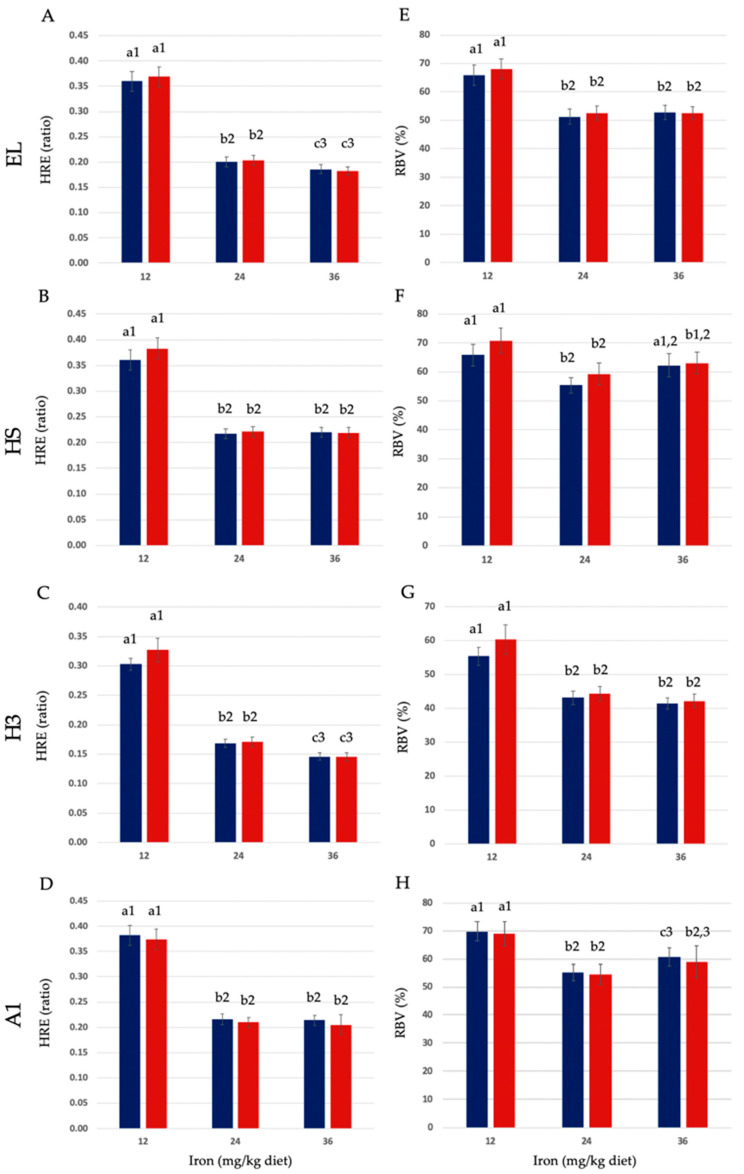
(**A**–**D**) Hemoglobin regeneration efficiency (HRE) and (**E**–**H**) relative iron bioavailability (RBV) of the EL (**A**,**E**), HS (**B**,**F**), H3 (**C**,**G**), and A1 (**D**,**H**) elemental iron powders (EIPs) in uncooked and cooked diets (blue and red bars, respectively). Values are mean ± SEM (n = 9–12/group). Different letters are used to denote significant differences (*p* ≤ 0.05) between different dietary iron (Fe) concentrations (mg Fe/kg diet) of the same treatment (uncooked and cooked). Different numbers are used to designate significant differences (*p* ≤ 0.05) between uncooked and cooked diets of the same Fe concentration. HRE ratio = [Final Hb Fe (mg) − Initial Hb Fe (mg)]/Fe intake (mg total consumed). RBV = Percentage (%) HRE relative to ferrous sulfate monohydrate (FS): HRE ratio of EIP/HRE ratio of the FS group × 100 (diet-matched).

Hemoglobin regeneration efficiency (HRE) and relative iron bioavailability (RBV) in anemic rats fed one of the four different elemental iron powders (EL, HS, H3, and A1) in uncooked or cooked diets are shown in Table 4, Table 5, Table 6 and Table 7, which also show HRE and RBV mean ± SEM for all diet groups. Food intake and, thus, iron intake and weight gain were positively associated with increasing dietary iron in all treatment groups (see Table 4, Table 5, Table 6 and Table 7). The HRE ratio and, thus, RBV calculations account for iron intake, and comparisons are diet-matched. HRE and RBV of each EIP in uncooked and cooked diets are shown in Figure 2A–H. For EL, the HRE ratios of uncooked diets at 12, 24, or 36 mg iron/kg diet (11.7, 23.5, and 36.2 mg iron/kg diet analyzed) were 0.360, 0.201, and 0.186, respectively (values ± SEM are shown in Table 4). The RBVs of iron from EL in uncooked diets at 12, 24, or 36 mg iron/kg diet were 65.8, 51.3, and 52.7, respectively. The HRE ratios of cooked diets containing EL at 12, 24, or 36 mg iron/kg diet (11.6, 24.3, and 35.8 mg iron/kg diet analyzed) were 0.369, 0.203, and 0.182, respectively. The RBVs of iron from EL in cooked diets at 12, 24, or 36 mg iron/kg diet were 68.1, 52.6, and 52.5, respectively. The overall mean RBVs (average RBV of the three dietary concentrations tested) of EL in the uncooked and cooked diets were 56.6% and 57.7%, respectively, and these did not differ significantly (*p* > 0.05). For HS, the HRE ratios of uncooked diets at 12, 24, or 36 mg iron/kg diet (12.3, 24.5, and 35.7 mg iron/kg diet analyzed) were 0.360, 0.217, and 0.220, respectively (Table 5). The RBVs of iron from HS in uncooked diets at 12, 24, or 36 mg iron/kg diet were 65.8, 55.4, and 62.3, respectively. The HRE ratios of cooked diets containing HS at 12, 24, or 36 mg iron/kg diet (11.8, 24.2, and 36.3 mg iron/kg diet analyzed) were 0.383, 0.220, and 0.219, respectively. The RBVs of iron from HS in these cooked diets at 12, 24, or 36 mg iron/kg diet were 70.7, 59.3, and 63.1, respectively. The overall mean RBVs (average of the three dietary concentrations tested) of HS in the uncooked and cooked diets were 61.2% and 64.4%, respectively, which were mildly significantly different (*p* ≤ 0.05). For H3, the HRE ratios of uncooked diets at 12, 24, or 36 mg iron/kg diet (12.2, 24.6, and 36.3 mg iron/kg diet analyzed) were 0.303, 0.169, and 0.146, respectively (Table 6). The RBVs of iron from H3 in uncooked diets at 12, 24, or 36 mg iron/kg diet were 55.4, 43.1, and 41.4, respectively. The HRE ratios of cooked diets containing H3 at 12, 24, or 36 mg iron/kg diet (11.7, 23.8, and 36.2 mg iron/kg diet analyzed) were 0.327, 0.171, and 0.146, respectively. The RBVs of iron from H3 in these cooked diets at 12, 24, or 36 mg iron/kg diet were 60.3, 44.3, and 42.1, respectively. The overall mean RBVs (average of the three dietary concentrations tested) of H3 in the uncooked and cooked diets were 46.6% and 48.9%, respectively, which were not significantly different (*p* > 0.05). For A1, the HRE ratios of uncooked diets at 12, 24, or 36 mg iron/kg diet (11.5, 23.6, and 35.7 mg iron/kg diet analyzed) were 0.382, 0.216, and 0.214, respectively (Table 7). The RBVs of iron from A1 in uncooked diets at 12, 24, or 36 mg iron/kg diet were 69.8, 55.1, and 60.6, respectively. The HRE ratios of cooked diets containing A1 at 12, 24, or 36 mg iron/kg diet (11.4, 24.4, and 36.3 mg iron/kg diet analyzed) were 0.374, 0.210, and 0.205, respectively. The RBVs of iron from A1 in cooked diets at 12, 24, or 36 mg iron/kg diet were 69.0, 54.4, and 59.1, respectively. The mean RBVs (average of the three dietary concentrations tested) of A1 in the uncooked and cooked diets were 61.8% and 60.8%, respectively, and these did not significantly differ (*p* > 0.05). HRE and RBV data showing mean ± SEM for all diet groups are shown in Table 4, Table 5, Table 6 and Table 7.

**Figure 3:**
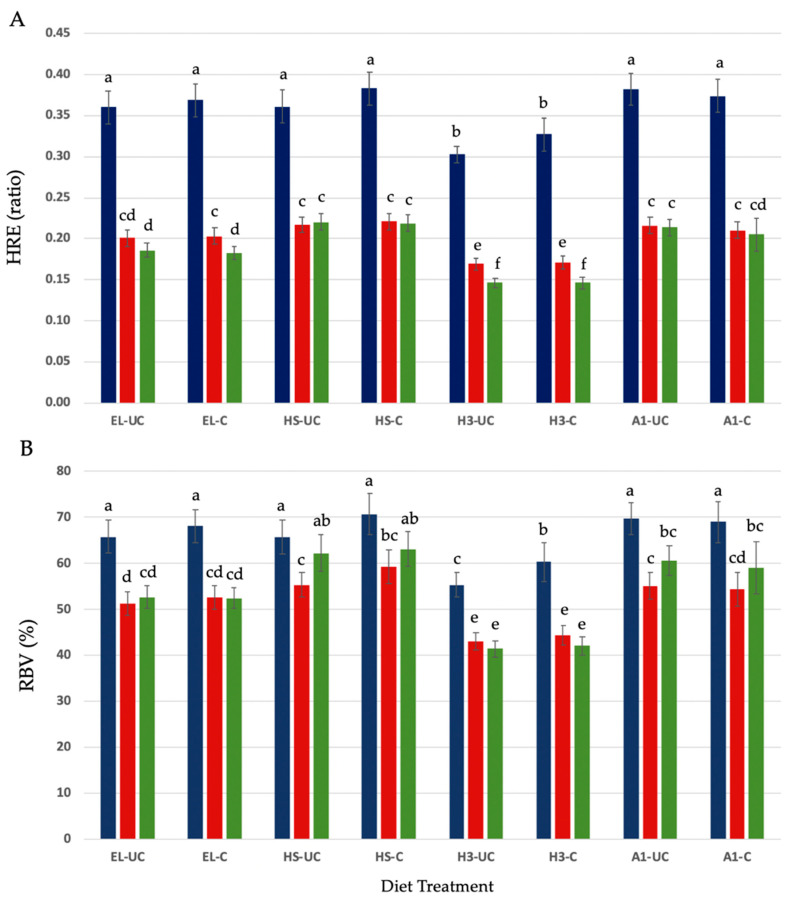
(**A**) Hemoglobin (Hb) regeneration efficiency (HRE) and (**B**) relative iron bioavailability (RBV) of the EL, HS, H3, and A1 elemental iron (Fe) powders (EIPs) in uncooked (UC) and cooked (C) diets, with blue, red, and green bars representing 12, 24, and 36 mg Fe/kg diet, respectively. Values are mean ± SEM (n = 9–12/group). Different letters are used to denote significant differences (*p* ≤ 0.05), from higher to lower HRE and RBV. HRE ratio = [Final Hb Fe (mg) − Initial Hb Fe (mg)]/Fe intake (mg total consumed). RBV is calculated as percentage of HRE relative to ferrous sulfate monohydrate (FS) = HRE ratio of EIP/HRE ratio of the FS group × 100 (diet-matched).

A combined comparison of all four EIPs’ HREs and RBVs is shown in Figure 3A,B.

### 3.4. Hemoglobin Maintenance Study

The ability of El-Lyte (EL) and Hi-Sol (HS) iron powders to maintain hemoglobin in non-anemic rats fed 6 mg iron/kg, uncooked and cooked diets, during a six-week period is shown in Table 8. EL and HS, at this concentration of dietary iron in uncooked and cooked diets, were both able to maintain hemoglobin levels as well as FS during the study period (mean ± SEM data for all groups are shown in Table 8).

## 4. Discussion

Our findings show that at the concentrations of iron tested, the EL, HS, H3, and A1 elemental iron powders are useful fortification agents to replenish hemoglobin and correct iron deficiency anemia. Data from this study uniquely illustrate the comparative HRE ratios and RBVs of these four elemental iron powders vs. ferrous sulfate when they are tested at different concentrations in uncooked and cooked diets. In the hemoglobin maintenance arm of this study, our data show that the EL and HS (less well-known elemental iron powders) were also able to maintain hemoglobin as well as ferrous sulfate at the same level of dietary iron (6 mg/kg diet) in non-anemic rats for six weeks. Iron dissolution testing in this study revealed that overall mean RBVs of the four elemental iron powders were positively correlated with iron solubility. However, the difference observed between RBV and percentage of iron dissolution in HS and A1 was greater than the difference seen with EL and H3. Similarly, other studies have shown a relationship between elemental iron powder dissolution properties and RBV, citing a relationship between RBV, particle size [27], and structure of the iron powders tested [32,33,34].

Generally, HREs and RBVs of these four elemental iron powders were significantly higher (*p* ≤ 0.05) at the lower concentration of dietary iron (12 mg Fe/kg diet) when compared to ferrous sulfate. Our data show that for RBV values, except for the H3 and HS samples tested at 12 mg Fe/kg diet—which had slightly higher overall mean RBVs with cooking (albeit non-significantly different; *p* > 0.05)—the effect of cooking is minimal, and cooking does not significantly (*p* ≤ 0.05) increase or decrease the RBVs of these elemental iron powders when in the type of dietary mixture used in this study. When compared to ferrous sulfate, the EL, HS, and A1 samples had >50% overall mean RBV, with the following general order of RBV: HS > A1 > EL > H3. The lower overall mean RBV seen with H3 in uncooked and cooked diets (at 46.6% and 48.9%, respectively) relates to the lower RBVs seen in this study at the 24 and 36 mg Fe/kg diet concentrations, which may be due to the differences seen when compared to ferrous sulfate at the higher levels of dietary iron tested.

The rat hemoglobin repletion assay has been used previously by micronutrient researchers worldwide, especially to study the ability of different types of iron fortificants to resolve iron deficiency anemia [6,27,28,32,33,35,36,37,38]. In the field of elemental iron powder research in animals and humans, there has been an effort to identify, optimize, and standardize the amount of dietary iron used [6,27,37,38,39]. Some have concluded that different dietary iron concentrations do not cause differential HRE values [6,30], while others claim there are noticeable differences in HRE at different dietary iron concentrations [39]. Our data here point more toward the conclusion that dietary iron concentration itself may affect HRE calculation, especially at lower iron concentrations. In this study, bakery-grade ferrous sulfate monohydrate was used as the positive control because iron from this compound (iron salt) has consistently been shown to be highly bioavailable [38,40], and it has been used in studies comparing different types of iron fortificants [32,34,38,41,42], ranging from the assessment of iron absorption in anemic school children [35] to adult humans [38,40,42] and rats [6,7,8,10,27,28,29], with most studies testing elemental iron powders in diets that were cooked, such as in cookies [42], or, more commonly, in wheat flours [32,33,35]. Additionally, although the monohydrate form of ferrous sulfate is less likely to form a crystalline microstructure, other iron salts used as oral iron supplements clinically (such as ferrous gluconate and ferrous fumarate) may under certain conditions precipitate into “particles” that may influence the absorption of iron from these compounds.

The mean RBV found in this study for EL (a type of electrolytic iron powder) in both uncooked and cooked diets is similar (within 5–15%) to the RBVs observed for other types of electrolytic iron powders, albeit those were tested in humans consuming iron-fortified bread rolls and compared to ferrous sulfate [43]. A study in humans using an H-reduced-elemental-iron-powder-fortified flour consumed by children 5–7 years of age found an RBV of approximately 65% [32], which is within 5–10% of the overall mean RBV we observed for EL, though well above the RBV we found for the H-reduced iron powder we tested (H3), depending on the diet group. However, the RBV we found for EL in this study is 5–15% higher than values summarized in a review [2] of RBVs of many previously tested elemental iron powders using rat hemoglobin repletion assays.

A human study testing a variety of elemental iron powders found that an H-reduced iron powder resulted in an RBV of 43%, while an electrolytic iron powder yielded an RBV of 73% [41]. The RBV of the H-reduced iron powder they tested is similar to the RBVs we found for the H-reduced iron powder we tested in this study (H3) when tested at the 24 and 36 mg Fe/kg diets but lower than the RBV of H3 at 12 mg Fe/kg diet, which may illustrate the influence that dietary iron concentration has when making comparative assessments between elemental iron powders and ferrous sulfate, especially at lower concentrations. The RBV of the electrolytic iron powder they used was very similar to our findings, being within 5–10% of the RBVs we determined for the elemental iron powders we tested at 12 mg Fe/kg diet, in both uncooked and cooked diets, with our cooked diets being more closely associated for each iron powder. However, for the EL, HS, and H3 iron powders we tested at 12 mg Fe/kg diet, the RBVs were slightly higher, although the differences were not significant (*p* > 0.05). Another study using rats, testing electrolytic, H-reduced, and CO-reduced iron elemental iron powders in comparison to ferrous sulfate found RBVs of 70%, 25%, and 32%, respectively [44], again highlighting the relatively lower RBV of H-reduced iron powders in comparison to electrolytic iron powders. Overall, our data are in agreement with the findings of a previous meta-analysis [2] and an international iron fortification guidelines review task force, which studied the usefulness of elemental iron for cereal flour fortification [45].

One limitation of this study may be that other cooking methods (i.e., dry vs. moist heat) were not tested. However, the cooking method we used in this study represents a standard baking approach and how a majority of manufactured food products using fortified and enriched cereal grain flours, into which these elemental iron powders are usually added, are treated in order to produce a variety of processed foods globally. Also, in the hemoglobin maintenance arm of this study, although serum ferritin was not measured, data on the ability of the two less-well-known elemental iron powders (EL and HS) to maintain iron in the functional compartment of blood (hemoglobin) without the rats becoming anemic during this period of time may be useful when considering new food fortification policies and guidelines, as other micronutrient programs have suggested [46].

Overall, data from this study show the relative efficacy of this group of four elemental iron powders in uncooked and cooked diets. Our findings indicate that an efficacious RBV may be achieved at somewhat lower rather than higher concentrations of dietary iron; this is important because current global iron fortification guidelines regarding elemental iron powders often suggest the addition of almost double the concentration of some types of elemental iron powders based on the typical approximate 50% RBV of many iron powders. Our findings support the efficacy of the EL, HS, and A1 elemental iron powders in fortified foods and as iron supplements in clinical settings in near-equivalent dietary iron concentrations to iron salts, such as ferrous sulfate. Based on the importance of resolving iron deficiency anemia worldwide [47], it is suggested that further studies and human clinical trials be aimed at determining what an optimal level of addition of each type of elemental iron powder may be and that prospective or relatively novel elemental iron powders, such as EL and HS, be taken forward for such trials under varying food processing and cooking conditions and in the presence of both dietary inhibitors and enhancers of non-heme iron absorption in order to maximize iron bioavailability when consumed with a mixture of foods.

## 5. Conclusions

In sum, these four elemental iron powders are useful fortification agents to replenish hemoglobin and correct iron deficiency anemia. Taking into consideration the favorable organoleptic profiles of elemental iron powders, inclusion of these elemental iron powders as iron fortificants in a variety of foods may be both efficacious and advantageous to reduce the incidence of iron deficiency and its associated anemia.

## Figures and Tables

**Table 1:** Baseline diet composition ^1^, from which treatment diets were prepared by adding iron (Fe, as ferrous sulfate monohydrate or one of the elemental iron powders).

**Formula**	**g/Kg**	
Casein, low Cu and Fe	200.0	
Sucrose	314.5	
Corn starch	315.0	
Soybean oil	70.0	
Cellulose, microcrystalline (Alphacel™)	50.0	
Mineral mix modified, no added iron (81062) ^2^ *	35.0	
Vitamin mix, AIN-93-VX (40077) ^3^ **	10.0	
L-cysteine	3.0	
Choline bitartrate	2.5	
TBHQ, antioxidant	0.014	
**Macronutrients**	**% dry weight**	**% kcal**
Protein	17.7	17.8
Carbohydrate	69.8	70.4
Fat	5.2	11.8

^1^ Ref: [26]. (The base AIN-93G[M] diet was prepared by Harlan Teklad (Catalog #TD.99397; Harlan Teklad, Madison, WI, USA)). ^2^ Calcium at 3.5 g/kg diet. * Atomic absorption spectrophotometry, performed in triplicate, was used to confirm iron concentrations of the diets, as previously described [27]). Without added iron, the baseline diet contained approximately 1.5 mg iron/kg diet. ^3^ Ascorbic acid at 200 mg/kg diet. ** Diet/ingredient catalog numbers shown; all other ingredients obtained from Harlan Teklad, Madison, WI, USA).

**Table 2:** Dissolution (solubility) of the four elemental iron powders ^1^.

Elemental Iron Powder	% Dissolution ^2,3^
El-Lyte	58.7 ± 1.1 ^a^
Hi-Sol	75.5 ± 2.7 ^b^
H-325	43.7 ± 1.4 ^c^
A-131	72.7 ± 3.0 ^b^

^1^ Determined at 30 min in HCl solution (pH 1.0) at 37 °C. ^2^ Values are mean ± SEM of triplicate assays. ^3^ Means in columns that do not share same letter are significantly different (*p* ≤ 0.05).

**Table 3:** Food and iron (Fe) intake and growth, hemoglobin change, and hemoglobin regeneration efficiency (HRE) in anemic rats fed no added iron (control) or bakery-grade ferrous sulfate monohydrate (FS) in uncooked (UC) or cooked (C) diets over a 14-day repletion period ^1,2^.

	Control (C) (No Added Iron)			Ferrous Sulfate (FS)								
Diet Code	C-UC	C-C	FS-1UC	FS-2UC	FS-3UC	FS-4UC	FS-5UC	FS-1C	FS-2C	FS-3C	FS-4C	FS-5C
Diet Fe (mg/kg)												
Calculated	1.6	1.6	6	12	18	24	36	6	12	18	24	36
(Analyzed)	(1.4)	(1.5)	(6.2)	(11.8)	(18.3)	(23.7)	(36.1)	(5.9)	(12.2)	(17.8)	(24.3)	(36.4)
Food intake (g/day)	11.8 ± 0.53 ^a^	11.9 ± 0.53 ^a^	12.1 ± 0.62 ^a^	12.9 ± 0.61 ^ab^	13.7 ± 0.73 ^bc^	14.6 ± 0.77 ^c^	14.9 ± 0.72 ^c^	12.3 ± 0.76 ^a^	13.1 ± 0.60 ^ab^	13.9 ± 0.75 ^bc^	14.5 ± 0.81 ^bc^	14.8 ± 0.80 ^c^
Fe intake (mg/day)	0.017 ± 7^−04 a^	0.018 ± 9^−04 a^	0.075 ± 4^−03 a^	0.152 ± 8^−03 b^	0.251 ± 0.01 ^c^	0.346 ± 0.02 ^d^	0.538 ± 0.02 ^e^	0.073 ± 4^−04 a^	0.160 ± 7^−03 b^	0.247 ± 0.01 ^c^	0.353 ± 0.02 ^d^	0.539 ± 0.03 ^e^
Body weight (g)												
Initial	84.1 ± 3.3 ^a^	83.8 ± 3.2 ^a^	82.1 ± 3.6 ^a^	83.4 ± 3.0 ^a^	83.9 ± 3.9 ^a^	84.3 ± 4.1 ^a^	84.7 ± 3.2 ^a^	83.2 ± 3.5 ^a^	84.1 ± 2.9 ^a^	83.6 ± 3.5 ^a^	84.4 ± 3.4 ^a^	83.9 ± 3.9 ^a^
(Gain)	(16.1 ± 0.6 ^a^)	(15.2 ± 0.5 ^a^)	(53.2 ± 2.3 ^a^)	(55.6 ± 2.7 ^a^)	(54.9 ± 2.5 ^a^)	(56.2 ± 2.3 ^a^)	(58.1 ± 2.9 ^a^)	(53.7 ± 2.3 ^a^)	(54.4 ± 2.6 ^a^)	(54.8 ± 2.9 ^a^)	(57.1 ± 2.0 ^a^)	(57.8 ± 3.2 ^a^)
Hemoglobin (g/dL)												
Initial	4.69 ± 0.2 ^a^	4.72 ± 0.4 ^a^	4.73 ± 0.2 ^a^	4.75 ± 0.3 ^a^	4.68 ± 0.2 ^a^	4.81 ± 0.3 ^a^	4.70 ± 0.2 ^a^	4.77 ± 0.2 ^a^	4.83 ± 0.3 ^a^	4.61 ± 0.4 ^a^	4.66 ± 0.3 ^a^	4.65 ± 0.3 ^a^
(Gain)	(−0.39 ± 0.02 ^a^)	(−0.41 ± 0.02 ^a^)	(1.24 ± 0.05 ^a^)	(1.83 ± 0.1 ^b^)	(3.36 ± 0.2 ^c^)	(4.10 ± 0.2 ^d^)	(6.39 ± 0.4 ^e^)	(1.29 ± 0.05 ^a^)	(2.01 ± 0.2 ^b^)	(3.14 ± 0.3 ^c^)	(4.09 ± 0.2 ^d^)	(6.33 ± 0.3 ^e^)
Hemoglobin Fe ^3^												
Gain (mg)	−0.082 ± 4^−03^ ^a^	−0.070 ± 3^−03^ ^b^	0.941 ± 0.03 ^a^	1.164 ± 0.04 ^b^	1.624 ± 0.03 ^c^	1.899 ± 0.09 ^d^	2.662 ± 0.14 ^e^	0.971 ± 0.03 ^a^	1.215 ± 0.04 ^b^	1.543 ± 0.08 ^c^	1.903 ± 0.11 ^d^	2.616 ± 0.15 ^e^
HRE ratio ^4^	n/a	n/a	0.896 ± 0.04 ^a^	0.547 ± 0.03 ^b^	0.462 ± 0.02 ^c^	0.392 ± 0.01 ^d^	0.353 ± 0.01 ^e^	0.950 ± 0.05 ^a^	0.542 ± 0.03 ^b^	0.446 ± 0.03 ^c^	0.386 ± 0.02 ^d^	0.347 ± 0.02 ^d^

^1^ Values are mean ± SEM (n = 9–12/group). ^2^ Means in same row of same diet type (i.e., UC or C) that do not share same letter(s) are significantly different (*p* ≤ 0.05). ^3^ Hb Fe (mg) = BW (body weight; kg) × 0.067 × Grams Hb per mL × 3.35 mg Fe. ^4^ HRE ratio = [Final Hb Fe (mg) − Initial Hb Fe (mg)]/Fe intake (mg total consumed).

**Table 4:** Food and iron (Fe) intake and growth, hemoglobin change, hemoglobin regeneration efficiency (HRE), and relative iron bioavailability (RBV) in anemic rats fed graded quantities of the El-Lyte (EL) elemental iron powder in uncooked (UC) or cooked (C) diets over a 14-day repletion period ^1,2^.

	El-Lyte (EL)					
Diet Code	EL-1UC	EL-2UC	EL-3UC	EL-1C	EL-2C	EL-3C
Diet Fe (mg/kg)						
Calculated	12	24	36	12	24	36
(Analyzed)	(11.7)	(23.5)	(36.2)	(11.6)	(24.3)	(35.8)
Food intake (g/day)	13.2 ± 0.62 ^a^	14.1 ± 0.76 ^ab^	14.9 ± 0.86 ^b^	12.9 ± 0.64 ^a^	13.9 ± 0.82 ^ab^	15.1 ± 0.91 ^b^
Fe intake (mg/day)	0.154 ± 7^−03 a^	0.331 ± 0.02 ^b^	0.539 ± 0.02 ^c^	0.150 ± 7^−03 a^	0.338 ± 0.02 ^b^	0.541 ± 0.03 ^c^
Body weight (g)						
Initial	84.3 ± 3.4 ^a^	83.6 ± 3.2 ^a^	84.9 ± 3.4 ^a^	83.8 ± 3.7 ^a^	85.1 ± 4.4 ^a^	84.6 ± 3.5 ^a^
(Gain)	(53.2 ± 2.6 ^a^)	(54.7 ± 2.1 ^a^)	(54.9 ± 3.3 ^a^)	(53.6 ± 1.9 ^a^)	(54.2 ± 3.1 ^a^)	(55.3 ± 2.7 ^a^)
Hemoglobin (g/dL)						
Initial	4.85 ± 0.4 ^a^	4.63 ± 0.2 ^a^	4.81 ± 0.3 ^a^	4.67 ± 0.2 ^a^	4.79 ± 0.2 ^a^	4.81 ± 0.3 ^a^
(Gain)	(0.64 ± 0.03 ^a^)	(1.17 ± 0.1 ^b^)	(2.59 ± 0.2 ^c^)	(0.69 ± 0.04 ^a^)	(1.20 ± 0.1 ^b^)	(2.48 ± 0.2 ^c^)
Hemoglobin Fe ^3^						
Gain (mg)	0.777 ± 0.03 ^a^	0.932 ± 0.03 ^b^	1.405 ± 0.05 ^c^	0.775 ± 0.04 ^a^	0.958 ± 0.04 ^b^	1.376 ± 0.06 ^c^
HRE ratio ^4^	0.360 ± 0.02 ^a^	0.201 ± 0.01 ^b^	0.186 ± 9^−03 c^	0.369 ± 0.02 ^a^	0.203 ± 0.01 ^b^	0.182 ± 8^−03 c^
RBV ^5^	65.8 ± 3.6 ^a^	51.3 ± 2.6 ^b^	52.7 ± 2.5 ^b^	68.1 ± 3.5 ^a^	52.6 ± 2.5 ^b^	52.5 ± 2.3 ^b^

^1^ Values are mean ± SEM (n = 9–12/group). ^2^ Means in same row of same diet type (i.e., UC or C) that do not share same letter(s) are significantly different (*p* ≤ 0.05). ^3^ Hb Fe (mg) = BW (body weight; kg) × 0.067 × Grams Hb per mL × 3.35 mg Fe. ^4^ HRE ratio = [Final Hb Fe (mg) − Initial Hb Fe (mg)]/Fe intake (mg total consumed). ^5^ RBV = Percentage (%) HRE relative to ferrous sulfate monohydrate (FS): HRE ratio of EL/HRE ratio of the FS group × 100 (diet-matched).

**Table 5:** Food and iron (Fe) intake and growth, hemoglobin change, hemoglobin regeneration efficiency (HRE), and relative iron bioavailability (RBV) in anemic rats fed graded quantities of the Hi-Sol (HS) elemental iron powder in uncooked (UC) or cooked (C) diets over a 14-day repletion period ^1,2^.

	Hi-Sol (HS)					
Diet Code	HS-1 UC	HS-2UC	HS-3UC	HS-1C	HS-2C	HS-3C
Diet Fe (mg/kg)						
Calculated	12	24	36	12	24	36
(Analyzed)	(12.3)	(24.5)	(35.7)	(11.8)	(24.2)	(36.3)
Food intake (g/day)	13.4 ± 0.53 ^a^	14.1 ± 0.65 ^ab^	15.2 ± 0.81 ^b^	13.3 ± 0.67 ^a^	13.9 ± 0.71 ^ab^	14.7 ± 0.59 ^b^
Fe intake (mg/day)	0.165 ± 7^−03 a^	0.345 ± 0.02 ^b^	0.543 ± 0.03 ^c^	0.157 ± 8^−03 a^	0.336 ± 0.02 ^b^	0.534 ± 0.03 ^c^
Body weight (g)						
Initial	84.7 ± 3.0 ^a^	84.9 ± 3.6 ^a^	85.2 ± 4.1 ^a^	84.1 ± 3.0 ^a^	85.3 ± 2.8 ^a^	85.1 ± 3.3 ^a^
(Gain)	(54.8 ± 2.6 ^a^)	(56.4 ± 2.9 ^a^)	(57.1 ± 2.2 ^a^)	(54.7 ± 2.1 ^a^)	(56.6 ± 3.4 ^a^)	(56.9 ± 2.6 ^a^)
Hemoglobin (g/dL)						
Initial	4.69 ± 0.3 ^a^	4.71 ± 0.2 ^a^	4.79 ± 0.3 ^a^	4.72 ± 0.2 ^a^	4.68 ± 0.2 ^a^	4.78 ± 0.3 ^a^
(Gain)	(0.81 ± 0.04 ^a^)	(1.43 ± 0.1 ^b^)	(3.31 ± 0.2 ^c^)	(0.84 ± 0.05 ^a^)	(1.51 ± 0.1 ^b^)	(3.22 ± 0.3 ^c^)
Hemoglobin Fe ^3^						
Gain (mg)	0.817 ± 0.04 ^a^	1.022 ± 0.04 ^b^	1.638 ± 0.07 ^c^	0.821 ± 0.03 ^a^	1.052 ± 0.05 ^b^	1.624 ± 0.06 ^c^
HRE ratio ^4^	0.361 ± 0.02 ^a^	0.217 ± 0.01 ^b^	0.220 ± 0.01 ^b^	0.383 ± 0.02 ^a^	0.221 ± 0.01 ^b^	0.219 ± 0.01 ^b^
RBV ^5^	65.8 ± 3.7 ^a^	55.4 ± 2.6 ^b^	62.3 ± 3.9 ^a^	70.7 ± 4.5 ^a^	59.3 ± 3.7 ^b^	63.1 ± 3.8 ^b^

^1^ Values are mean ± SEM (n = 9–12/group). ^2^ Means in same row of same diet type (i.e., UC or C) that do not share same letter(s) are significantly different (*p* ≤ 0.05). ^3^ Hb Fe (mg) = BW (body weight; kg) × 0.067 × Grams Hb per mL × 3.35 mg Fe. ^4^ HRE ratio = [Final Hb Fe (mg) − Initial Hb Fe (mg)]/Fe intake (mg total consumed). ^5^ RBV = Percentage (%) (FS): HRE ratio of HS/HRE ratio of the FS group × 100 (diet-matched).

**Table 6:** Food and iron (Fe) intake and growth, hemoglobin change, hemoglobin regeneration efficiency (HRE), and relative iron bioavailability (RBV) in anemic rats fed graded quantities of the H-325 (H3) elemental iron powder in uncooked (UC) or cooked (C) diets over a 14-day repletion period ^1,2^.

	H-325 (H3)					
Diet Code	H3-1UC	H3-2UC	H3-3UC	H3-1C	H3-2C	H3-3C
Diet Fe (mg/kg)						
Calculated	12	24	36	12	24	36
(Analyzed)	(12.2)	(24.6)	(36.3)	(11.7)	(23.8)	(36.2)
Food intake (g/day)	13.1 ± 0.49 ^a^	14.6 ± 0.66 ^b^	15.1 ± 0.71 ^b^	12.8 ± 0.67 ^a^	14.1 ± 0.73 ^ab^	14.7 ± 0.62 ^b^
Fe intake (mg/day)	0.160 ± 8^−03 a^	0.359 ± 0.02 ^b^	0.548 ± 0.03 ^c^	0.150 ± 9^−03 a^	0.336 ± 0.05 ^b^	0.532 ± 0.04 ^c^
Body weight (g)						
Initial	84.8 ± 4.3 ^a^	83.7 ± 3.6 ^a^	84.6 ± 3.2 ^a^	83.2 ± 3.1 ^a^	84.1 ± 3.8 ^a^	83.9 ± 4.1 ^a^
(Gain)	(50.8 ± 2.5 ^a^)	(51.2 ± 2.1 ^a^)	(51.9 ± 3.1 ^a^)	(51.6 ± 3.0 ^a^)	(51.0 ± 2.1 ^a^)	(52.6 ± 2.2 ^a^)
Hemoglobin (g/dL)						
Initial	4.68 ± 0.2 ^a^	4.82 ± 0.3 ^a^	4.71 ± 0.3 ^a^	4.73 ± 0.3 ^a^	4.65 ± 0.5 ^a^	4.73 ± 0.3 ^a^
(Gain)	(0.48 ± 0.03 ^a^)	(0.98 ± 0.1 ^b^)	(1.86 ± 0.3 ^c^)	(0.46 ± 0.03 ^a^)	(0.89 ± 0.1 ^b^)	(1.72 ± 0.2 ^c^)
Hemoglobin Fe ^3^						
Gain (mg)	0.679 ± 0.03 ^a^	0.851 ± 0.04 ^b^	1.119 ± 0.04 ^c^	0.687 ± 0.03 ^a^	0.802 ± 0.02 ^b^	1.085 ± 0.04 ^c^
HRE ratio ^4^	0.303 ± 0.01 ^a^	0.169 ± 7^−03 b^	0.146 ± 6^−03 c^	0.327 ± 0.02 ^a^	0.171 ± 8^−03 b^	0.146 ± 7^−03 c^
RBV ^5^	55.4 ± 2.7 ^a^	43.1 ± 1.9 ^b^	41.4 ± 1.7 ^b^	60.3 ± 4.3 ^a^	44.3 ± 2.1 ^b^	42.1 ± 2.0 ^b^

^1^ Values are mean ± SEM (n = 9–12/group). ^2^ Means in same row of same diet type (i.e., UC or C) that do not share same letter(s) are significantly different (*p* ≤ 0.05). ^3^ Hb Fe (mg) = BW (body weight; kg) X 0.067 X Grams Hb per mL X 3.35 mg Fe. ^4^ HRE ratio = [Final Hb Fe (mg) − Initial Hb Fe (mg)]/Fe intake (mg total consumed). ^5^ RBV = Percentage (%) HRE relative to ferrous sulfate monohydrate (FS): HRE ratio of H3/HRE ratio of the FS group × 100 (diet-matched).

**Table 7:** Food and iron (Fe) intake and growth, hemoglobin change, hemoglobin regeneration efficiency (HRE), and relative iron bioavailability (RBV) in anemic rats fed graded quantities of the A-131 (A1) elemental iron powder in uncooked (UC) or cooked (C) diets over a 14-day repletion period ^1,2^.

	A-131 (A1)					
Diet Code	A1-1UC	A1-2UC	A1-3UC	A1-1C	A1-2C	A1-3C
Diet Fe (mg/kg)						
Calculated	12	24	36	12	24	36
(Analyzed)	(11.5)	(23.6)	(35.7)	(11.4)	(24.4)	(36.3)
Food intake (g/day)	13.3 ± 0.70 ^a^	13.8 ± 0.59 ^ab^	14.8 ± 0.82 ^b^	12.7 ± 0.58 ^a^	13.7 ± 0.53 ^a^	15.2 ± 0.92 ^b^
Fe intake (mg/day)	0.153 ± 8^−03 a^	0.326 ± 0.02 ^ab^	0.528 ± 0.02 ^b^	0.145 ± 7^−03 a^	0.334 ± 0.02 ^a^	0.552 ± 0.03 ^b^
Body weight (g)						
Initial	84.2 ± 2.9 ^a^	84.5 ± 3.0 ^a^	84.3 ± 5.5 ^a^	83.6 ± 3.3 ^a^	84.6 ± 2.8 ^a^	84.3 ± 3.9 ^a^
(Gain)	(54.2 ± 3.1 ^a^)	(55.2 ± 2.7 ^a^)	(56.7 ± 2.4 ^a^)	(53.9 ± 2.4 ^a^)	(55.9 ± 2.6 ^a^)	(56.7 ± 3.2 ^a^)
Hemoglobin (g/dL)						
Initial	4.71 ± 0.3 ^a^	4.75 ± 0.2 ^a^	4.61 ± 0.2 ^a^	4.69 ± 0.2 ^a^	4.84 ± 0.3 ^a^	4.70 ± 0.3 ^a^
(Gain)	(0.79 ± 0.04 ^a^)	(1.27 ± 0.05 ^b^)	(3.06 ± 0.3 ^c^)	(0.64 ± 0.03 ^a^)	(1.19 ± 0.05 ^b^)	(3.12 ± 0.2 ^c^)
Hemoglobin Fe ^3^						
Gain (mg)	0.818 ± 0.04 ^a^	0.987 ± 0.04 ^b^	1.581 ± 0.06 ^c^	0.759 ± 0.03 ^a^	0.983 ± 0.04 ^b^	1.586 ± 0.07 ^c^
HRE ratio ^4^	0.382 ± 0.02 ^a^	0.216 ± 0.01 ^b^	0.214 ± 0.01 ^b^	0.374 ± 0.02 ^a^	0.210 ± 0.01 ^b^	0.205 ± 0.02 ^b^
RBV ^5^	69.8 ± 3.5 ^a^	55.1 ± 2.9 ^b^	60.6 ± 3.3 ^c^	69.0 ± 4.4 ^a^	54.4 ± 3.6 ^b^	59.1 ± 5.7 ^b^

^1^ Values are mean ± SEM (n = 9–12/group). ^2^ Means in same row of same diet type (i.e., UC or C) that do not share same letter(s) are significantly different (*p* ≤ 0.05). ^3^ Hb Fe (mg) = BW (body weight; kg) X 0.067 X Grams Hb per mL X 3.35 mg Fe. ^4^ HRE ratio = [Final Hb Fe (mg) − Initial Hb Fe (mg)]/Fe intake (mg total consumed). ^5^ RBV = Percentage (%) HRE relative to ferrous sulfate monohydrate (FS): HRE ratio of A1/HRE ratio of the FS group × 100 (diet-matched).

**Table 8:** Ability of El-Lyte (EL) and Hi-Sol (HS) iron powders to maintain hemoglobin in non-anemic rats fed 6 mg iron/kg, uncooked (UC) and cooked (C) diets, during six-week period ^1,2^.

	Control (No Added Iron)		Ferrous Sulfate (FS)		EL-Lyte (EL)		Hi-Sol (HS)	
Diet Code	C-UC	C-C	FS-UC	FS-C	EL-UC	EL-C	HS-UC	HS-C
Diet Fe (mg/kg)								
Calculated	1.6	1.6	6	6	6	6	6	6
(Analyzed)	(1.4)	(1.5)	(6.2)	(6.1)	(5.8)	(6.2)	(6.1)	(5.9)
Hemoglobin (g/dL)								
Baseline (start wk 0)	13.2 ± 0.5 ^a^	12.9 ± 0.6 ^a^	12.8 ± 0.9 ^a^	13.1 ± 0.7 ^a^	13.4 ± 0.8 ^a^	13.0 ± 0.7 ^a^	13.3 ± 0.9 ^a^	12.9 ± 0.6 ^a^
Mid (end wk 3)	5.3 ± 0.3 ^a^	4.9 ± 0.4 ^a^	12.6 ± 0.8 ^b^	12.4 ± 0.6 ^b^	12.1 ± 0.5 ^b^	12.0 ± 0.4 ^b^	12.8 ± 0.9 ^b^	12.6 ± 0.3 ^b^
Post (end wk 6)	4.1 ± 0.3 ^a^	4.2 ± 0.2 ^a^	12.9 ± 0.8 ^b^	12.8 ± 0.7 ^b^	12.3 ± 0.9 ^b^	12.1 ± 0.6 ^b^	12.4 ± 0.4 ^b^	12.7 ± 0.5 ^b^

^1^ Values are mean ± SEM (n = 9–12/group). ^2^ Means in same row with letter(s) that differ are significantly different (*p* ≤ 0.05).

## Data Availability

The original contributions and data based on which the findings are presented in this study are included within the article and the Supporting Material that accompany this submission.

## Supplementary Materials

The following supporting information can be downloaded at: https://www.mdpi.com/article/10.3390/nu16142258/s1. Figure S1: Dissolution (% solubility) of the EL, HS, H3, and A1 elemental iron powders determined at 30 min in HCl solution (pH 1.0) at 37 °C. Figure S2: Hemoglobin change (g/dL) in the control (no added iron) and ferrous sulfate monohydrate (FeSO_4_•H_2_O) uncooked and cooked diets.
