# Supplementary material for: Hemoglobin Regeneration Efficiency and Relative Iron Bioavailability of Four Elemental Iron Powders in Rats
Source: Nutrients. 2024 Jul 13;16(14):2258. doi: 10.3390/nu16142258 (PMC11279874; doi:10.3390/nu16142258)
Supplement: Supplementary file 1 [file nutrients-16-02258-s001.zip › nutrients-3103753-supplementary.pdf]

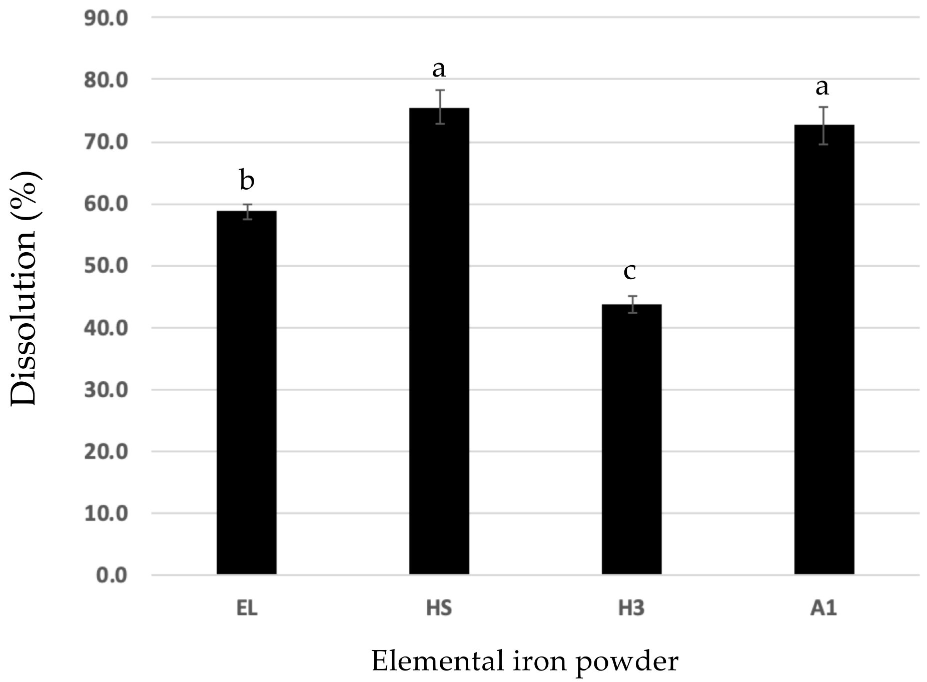

Figure S1. Dissolution (% solubility) of the EL, HS, H3, and A1 elemental iron powders determined at 30 min in HCl solution (pH 1.0) at 37°C. Values are mean ± SEM of triplicate assays. Different letters are used to denote significant differences ($p \leq 0.05$), from higher to lower dissolution.

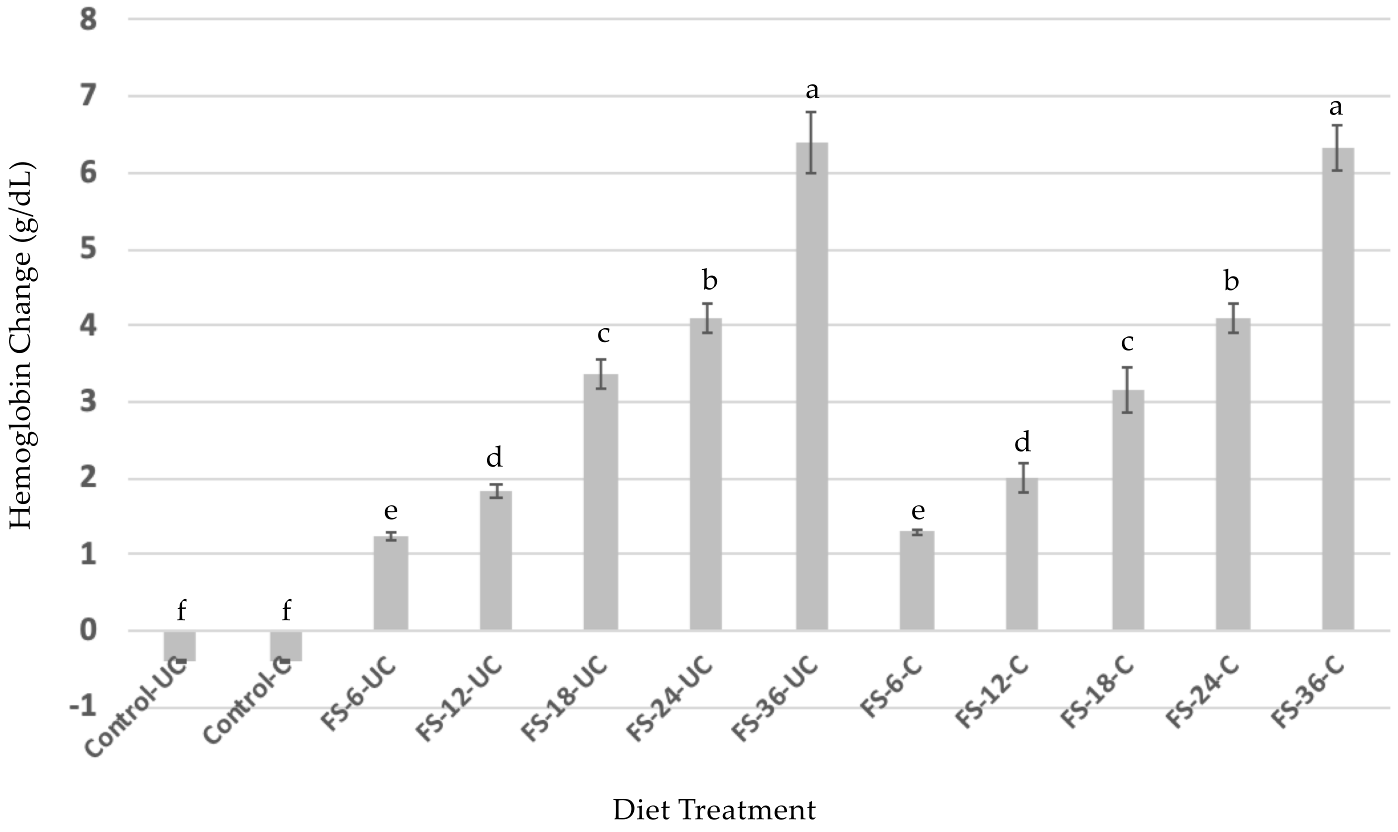

Figure S2. Hemoglobin change (g/dL) in the control (no added iron) and ferrous sulfate monohydrate ($FeSO_4 \bullet H_2O$; FS) uncooked (UC) and cooked (C) diets. Values are mean ± SEM (n=9-12/group). Different letters are used to denote significant differences ($p \leq 0.05$), from higher to lower hemoglobin change.
